# Simultaneous regeneration of full-thickness cartilage and subchondral bone defects *in vivo* using a three-dimensional scaffold-free autologous construct derived from high-density bone marrow-derived mesenchymal stem cells

**DOI:** 10.1186/s13018-014-0098-z

**Published:** 2014-10-14

**Authors:** Kohei Ishihara, Koichi Nakayama, Shizuka Akieda, Shuichi Matsuda, Yukihide Iwamoto

**Affiliations:** Department of Orthopaedic Surgery, Graduate School of Medical Sciences, Kyushu University, 3-1-1 Maidashi, Higashi-ku, Fukuoka City, Fukuoka 812-8582 Japan; Department of Advanced Technology Fusion, Graduate School of Science and Engineering, Saga University, 1 Honjo-machi, Saga City, Saga 840-8502 Japan

**Keywords:** Tissue engineering, Mesenchymal stem cells, Osteochondral cartilage defect, Scaffold-free, Cartilage repair

## Abstract

**Background:**

In recent years, several methods have been developed for repairing full-thickness cartilage defects by tissue engineering using mesenchymal stem cells. Most of these use scaffolds to achieve sufficient thickness. However, considering the potential influence of scaffolds on the surrounding microenvironment, as well as immunological issues, it is desirable to develop a scaffold-free technique. In this study, we developed a novel technique, a scaffold-free autologous construct derived from bone marrow-derived mesenchymal stem cells (BM-MSCs), and successfully use this technique to regenerate cartilage and subchondral bone to repair an osteochondral defect in rabbit knees.

**Methods:**

BM-MSCs were isolated from bone marrow liquid aspirated from the iliac crest of rabbits. After expansion in culture dishes and re-suspension in 96-well plates, the cells spontaneously aggregated into a spheroid-like structure. The spheroids were loaded into a tube-shaped Teflon mold with a 5-mm height and maintained under air-liquid interface conditions. These loaded spheroids fused with each other, resulting in a cylinder-shaped construct made of fused cells that conformed to the inner shape of the mold. The construct was implanted into an osteochondral defect in rabbit knees and histologically analyzed 24 and 52 weeks after implantation using Wakitani’s scoring system.

**Results:**

Both bone and cartilage were regenerated, maintaining a constant thickness of cartilage. The mean histological score was 10 ± 1.7 in the 24-week group and 9.7 ± 0.6 in the 52-week group. There was no significant difference between the 24- and 52-week groups in either parameter of the score, indicating that no deterioration of the repaired tissue occurred during the intervening period.

**Conclusions:**

Using our novel technique, which employs a three-dimensional scaffold-free autologous construct derived from BM-MSCs, we successfully achieved simultaneous regeneration of bone and cartilage for up to 1 year *in vivo*. This method has potential for clinical use as a safe and effective method for repairing bone and cartilage defects.

## Background

Repairing damaged cartilage, as in traumatic lesions of the joint surface or degenerative damage due to osteoarthritis, remains a clinical challenge because of the poor self-healing ability of cartilage [1]. Several existing treatments for the repair of damaged cartilage, including mosaicplasty [2], microfracture [3], and autologous chondrocyte implantation (ACI) [4], have been used successfully in patients to relieve pain and improve joint function. However, the long-term results of mosaicplasty and microfracture are unsatisfactory because the replaced tissue is not physiological hyaline cartilage, but mostly fibrous cartilage, which lacks suitable mechanical properties [5,6]. Furthermore, in histological evaluations of the repaired tissue, ACI exhibited no significant difference compared to microfracture in a randomized trial [7], and the repaired tissue was predominantly fibrous cartilage.

Tissue engineering of functional articular cartilage could help address these issues. In recent years, several methods have been developed to repair full-thickness cartilage defects. Some of these methods use synthetic or biological scaffolds to achieve sufficient thickness, mechanical function, support cell attachment, migration, proliferation, and differentiation [8,9]. However, these materials can influence the surrounding microenvironments [10] or cause immunological problems [11,12].

Several scaffold-free systems have also been investigated [13-16]. Without using a scaffold, however, it is difficult to create enough thickness to fill in the defect. A thickness of 5 mm is necessary to fill a full-thickness articular cartilage defect in the knee. Furthermore, with or without using a scaffold, it is even more challenging for the current methods to regenerate both articular cartilage and subchondral bone in the context of an osteochondral defect, which sometimes occurs in trauma, osteochondritis dissecans, osteonecrosis, or osteoarthritis.

Adherent cells grown in suspension form spheroids (aggregates) in order to avoid cell death. We confirmed this phenomenon using bone marrow-derived mesenchymal stem cells (BM-MSCs). Furthermore, we developed a novel method for fusing spheroids derived from BM-MSCs to make a large construct without using a scaffold. Loading the spheroids into a molding chamber can produce a columnar structure consisting of fused spheroids; we named this structure “high-density mesenchymal stem cell, scaffold-free autologous construct” (HDMAC). We hypothesized that HDMACs could be used to regenerate both articular cartilage and subchondral bone in the context of an osteochondral defect. Furthermore, because many other reports have achieved successful short-term cartilage repair, we focused on achieving a relatively long-term result (up to 1 year) in an animal model.

The purposes of this study were to demonstrate our method for making spheroids and a scaffold-free construct (HDMAC) using only undifferentiated BM-MSCs and to evaluate the 1-year outcome of simultaneous regeneration of cartilage and subchondral bone in an osteochondral defect in rabbit knees.

## Materials and methods

### Rabbits

All experiments followed protocols approved by the institutional animal care and use committee (approval number: A21-204-2). Fifteen mature female Japanese white rabbits (Japan Kyudo Inc, Tosu, Japan) with a mean weight of 3.9 kg (range 3.5–4.2) were eventually used for this study after several pilot studies to establish the procedures in detail. Animals were housed in separate cages at the animal center of our institution with free access to water and standard food. Two or three rabbits were euthanized at 3, 6, and 12 weeks and three rabbits were euthanized at each of two time points (24 and 52 weeks) after implantation for histological analysis.

### Culture medium

Dulbecco’s modified Eagle’s medium (DMEM) (Nissui #05915, Nissui Pharmaceutical, Tokyo, Japan) containing 10% fetal bovine serum (FBS) (HyClone, Logan, UT, USA), 100 U/mL penicillin, and 100 μg/mL streptomycin (Sigma-Aldrich, St. Louis, MO, USA) was used. No differentiation agents were used from cell isolation to construct formation.

### Isolation and culture of mesenchymal stem cells (MSCs)

MSCs were isolated as described previously with some modifications in the method [9]. Briefly, bone marrow liquid was aspirated from the iliac crest of the rabbits under general anesthesia, and the obtained bone marrow was mixed with heparin. After centrifugation at 1,000 rpm for 5 min, the buffy coat of the bone marrow liquid was aspirated and expanded into a 15-cm culture dish containing DMEM and 10% FBS. The cells obtained were expanded until they reached the necessary numbers (~4 × 10^7^ cells).

### Spheroid formation

**Figure 1:**
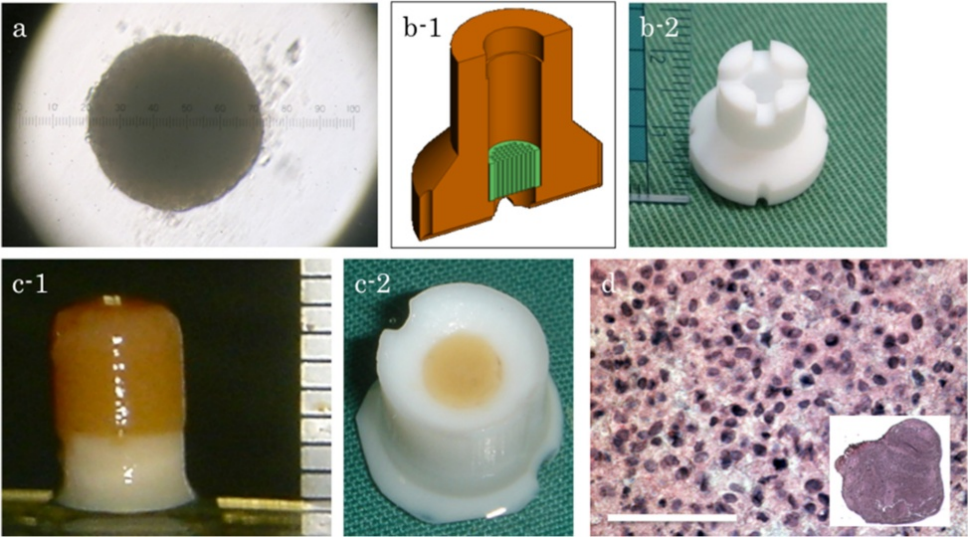
**Manufacturing HDMACs. (a)** BM-MSCs were isolated from bone marrow liquid aspirated from rabbits. After expansion, re-suspension, and incubation, the cells spontaneously aggregated into a spheroid-like structure. Each scale unit represents 1 μm. **(b)** A handmade tube-shaped Teflon mold with a diameter of 4.6 mm and a height of 5 mm. The spheroids described in **(a)** were loaded into this mold. Each scale unit represents 1 mm. **(c)** The tube-shaped mold was maintained under air-liquid interface conditions and incubated for a week. These loaded spheroids fused with each other, and a cylinder-shaped construct made with fused cells was obtained according to the inner shape of the mold. Each scale unit represents 1 mm. **(d)** Safranin O staining of HDMACs after 1 week of incubation. Scale bar =100 μm. (*N* = 1).

Cells were detached by recombinant trypsin replacement, re-suspended in DMEM plus 10% FBS, and then divided into 96-well plates designed to prevent cells from adhering to the culture surface (Sumilon PrimeSurface, Sumitomo Bakelite, Tokyo, Japan) at 4 × 10^4^ cells per well. After incubation at 37°C and 5% CO_2_ for 2 days, the cells spontaneously aggregated into a spheroid-like structure (Figure 1a).

### Construct formation

The spheroids described above were loaded into a handmade tube-shaped Teflon mold with a diameter of 4.6 mm and a height of 5 mm (Figure 1b). At the bottom of the mold, a base disc was placed into the chamber; the base disc was used for handling of the construct. Six hundred to seven hundred spheroids were needed to make one construct. The tube-shaped mold was maintained under air-liquid interface conditions and incubated at 37°C and 5% CO_2_ for 1 week in DMEM plus 10% FBS.

These loaded spheroids fused with each other, resulting in formation of a cylinder-shaped construct made of fused cells that conformed to the inner shape of the mold (Figure 1c). We named these structures “high-density mesenchymal stem cell, scaffold-free autologous constructs” (HDMACs).

### Scanning electron microscopy (SEM)

Constructs were fixed with 1% glutaraldehyde/1.44% paraformaldehyde in buffer at 37°C for 60 min, frozen in liquid nitrogen, and lyophilized. Next, samples were sputter-coated with an alloy of platinum and palladium and then observed by scanning electron microscopy (JSM 840A, JEOL USA, Peabody, MA, USA).

### Implantation *in vivo*

After general anesthesia, a cylindrical defect was created in the articular cartilage of the patella groove. The diameter of the defect was set to 4.8 mm. The depth was determined to be the same as the height of the cell construct, usually 4–5 mm in depth, which reached the middle layer of the subchondral bone. The construct was gently implanted into the defect, the Teflon disc was removed, and the top of the construct was flattened using the surgeon’s finger. No patches were placed on the implant surface. The osteochondral defect in the contralateral knee was left empty for controls. After washing and closure of the wound, the knee was immobilized with a cast for 1 week. In a pilot study, individual rabbits were euthanized at 3, 6, and 12 weeks to confirm the early regeneration process by histological evaluation. After obtaining positive results from this pilot study, three knees were obtained at each of two time points (24 and 52 weeks) and subjected to histological analysis.

### Micro-CT scanning

For morphological observation, the specimens were scanned using a micro-CT (Hitachi-Aloka Medical, Tokyo, Japan) after euthanasia. Sliced CT data were three-dimensionally reconstructed (Real INTAGE, Kubota Graphics Technology, Tokyo, Japan).

### Histological analysis and immunohistochemistry

Specimens were fixed in 4% paraformaldehyde, decalcified with 0.5 M ethylenediaminetetraacetic acid (EDTA), and embedded in paraffin wax. Sagittal sections (5 μm thick) were stained with safranin O and Weigert’s iron hematoxylin and then examined by light microscopy. For immunohistochemistry, sections were pretreated with 0.4 mg/mL proteinase K (DAKO, Carpinteria, CA, USA) for 10 min at room temperature. Endogenous peroxidases were quenched using 3% hydrogen peroxide in methanol for 20 min at room temperature. The sections were then incubated with mouse polyclonal antibody against type II collagen (F-57 anti-hCL (II), FUJI Chemical Industries, Toyama, Japan) and rabbit polyclonal antibody against type X collagen (ab58632, Abcam, Cambridge, MA, USA) at room temperature for 1 h. The immunostaining was performed using the VECTASTAIN ABC reagent (Vector Laboratories, Burlingame, CA, USA) and developed in 3,3’-diaminobenzidine and 0.02% H_2_O_2_ with hematoxylin counterstaining.

### Histological scoring

Histological findings were scored according to a scale described by Wakitani et al. [9]. Sections were graded according to 1) cell morphology (maximum 4 points), 2) matrix staining (maximum 3 points), 3) surface regularity (maximum 3 points), 4) thickness of cartilage (maximum 2 points), and 5) integration of donor material into adjacent host cartilage (maximum 2 points). Scoring was performed blindly by two orthopedic surgeons who were independent of this study, and the average of each score was adopted.

### Cell tracking

At first passage, cells were re-suspended at in DMEM 1.0 × 10^6^ cells/mL and then were labeled with Qtracker 655 Cell Labeling Kit (Quantum Dot Corp., Hayward, CA, USA).

The day after the cell labeling, the cells proceeded to spheroid formation as described above. HDMACs generated using the labeled cells were implanted in rabbit knees as described above, and the rabbits were 6 weeks later for histological examination.

### Statistical analysis

For *in vivo* investigations, nonparametric comparisons were performed using Student’s *t* test. *P* values less than 0.05 were considered to represent statistical significance.

## Results

### Histology and scanning electron micrographs of HDMACs

**Figure 2:**
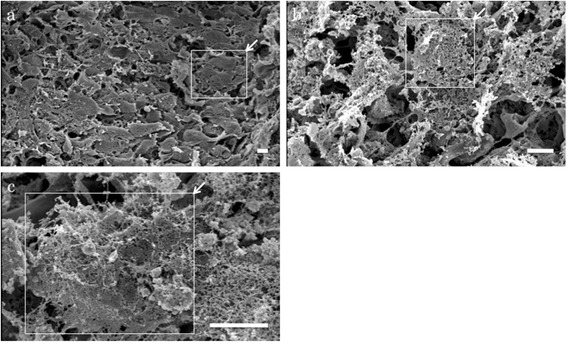
**Scanning electron micrographs of HDMACs.** SEMs showed that these constructs contained flattened cells (arrows and frames) with some amount of collagen fibers. Original magnifications were **(a)** 430×, **(b)** 1,000×, and **(c)** 2,500×. Scale bar =10 μm. (*N* = 1).

Histological examination of HDMACs with safranin O staining after 1 week of incubation revealed that the nucleus of cells were stained without fragmentation or chromatin condensation at the whole area of the section, suggesting that the cells in the construct were viable and did not undergo apoptosis (Figure 1d). The matrix was not stained red, suggesting that the cells in the HDMACs had not been differentiated into chondrocytes before the implantation. The ultrastructural morphology of the HDMACs was assessed by SEM. The micrographs revealed that these constructs contained flattened cells with some amount of collagen fibers (Figure 2).

### Pilot study for early repairing process in osteochondral defect

**Figure 3:**
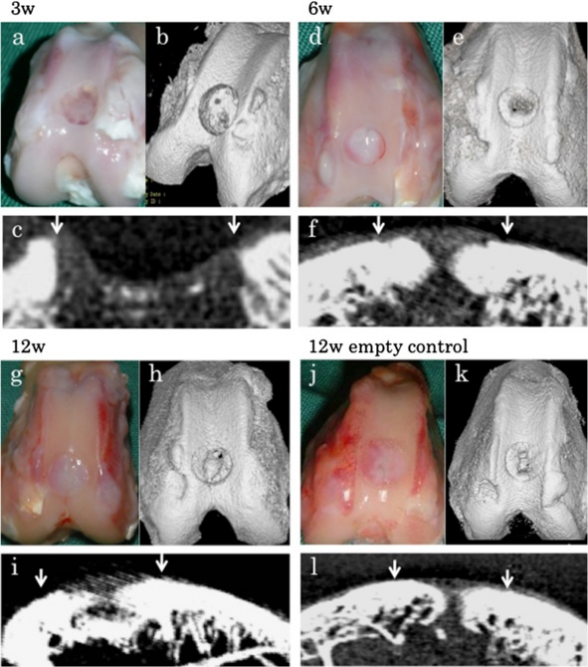
**Early repair processes in osteochondral defects.** Gross appearance at **(a)** 3 weeks, **(d)** 6 weeks, and **(g)** 12 weeks. After 6 weeks of implantation, the surface of the defect was covered with cartilaginous white tissue. Micro-CT scanning at **(b, c)** 3 weeks, **(e, f)** 6 weeks, and **(h, i)** 12 weeks suggested that bone formations were present at the peripheral areas and developed toward the central area. **(j)** Gross appearance and **(k, l)** micro-CT scanning of a control rabbit without any implantation at 12 weeks. Although the gross appearance showed that the defect was filled with white tissues, the subchondral bone formation was incomplete at the center of the defect at 12 weeks. Downwards arrows indicate the borders of the defect. Representative subjects of two or three experiments for each time course are shown.

**Figure 4:**
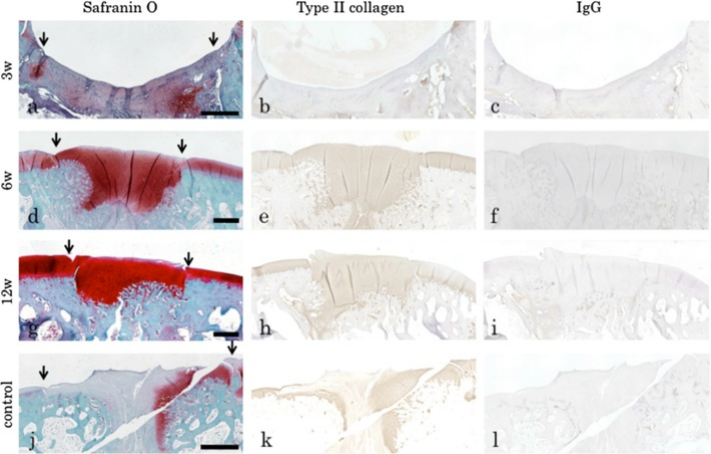
**Histological examination at early repair processes in osteochondral defects.** Safranin O staining, immunostaining for type II collagens, and IgG staining as controls at **(a–c)** 3 weeks, **(d–f)** 6 weeks, and **(g–i)** 12 weeks after the implantation. At 3 weeks, the implanted tissue had not differentiated into hyaline cartilage yet. At 6 and 12 weeks, the joint surface area of the implanted tissue was stained with safranin O and immunostained for type II collagen. Endochondral bone formation was observed at the peripheral area at 6 weeks and developed toward the central area at 12 weeks. **(j–l)** A control rabbit without any implantation at 12 weeks. The defect was filled with fibrous tissue that did show a constant staining for safranin O and immunostaining for type II collagen, especially at the surface and central areas. Scale bar = 1 mm. Downwards arrows indicate the borders of the defect. Representative subjects of two or three experiments for each time course are shown.

To test our hypothesis that HDMACs would be able to simultaneously regenerate cartilage and bone, two or three rabbits were euthanized at 3, 6, and 12 weeks and morphologically and histologically assessed (Figures 3a–i and 4a–i). After 6 weeks of implantation, the surface of the defect was covered with cartilaginous white tissue. Micro-CT scanning also suggested that bone formations were present at the peripheral areas and developed toward the central area. Histological examination suggested that cartilaginous differentiation, which was detected by safranin O staining and immunostaining for type II collagens, occurred at peripheral areas of the implanted HDMACs and developed toward the central area. At 6 weeks, all the implanted area showed positive staining for safranin O and type II collagen. At 12 weeks, endochondral bone formation progressed, and almost the whole area corresponding to the subchondral bone was replaced by bone structures. At the joint surface area of the implanted tissue, hyaline cartilage was regenerated with a constant thickness.

By contrast, in a control rabbit without any implantation, the defect was filled with fibrous tissue. Although a spontaneous repairing process was observed at the peripheral area that showed slight staining for safranin O or type II collagen, the cartilage formation and the subchondral bone formation were incomplete at the central area even at 12 weeks (Figures 3j–l and 4j–l).

### Cell tracking

**Figure 5:**
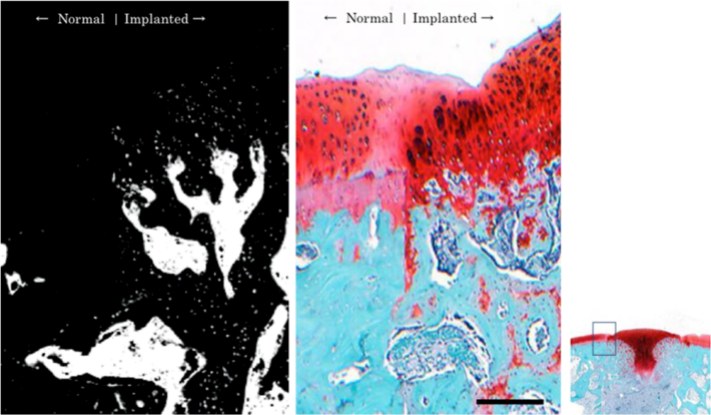
**Cell tracking.** HDMACs made with labeled MSCs were implanted to the osteochondral defect. The labeled cells were detected as chondrocytes in the cartilage and osteocytes in the subchondral bone 6 weeks after implantation, suggesting that the implanted MSCs had spontaneously differentiated into chondrocytes or osteocytes in accordance with their implanted positions. Scale bar = 200 μm. (*N* = 1).

HDMACs were made with labeled MSCs and implanted into the osteochondral defect. Labeled cells were detected as chondrocytes in the cartilage and osteocytes in the subchondral bone 6 weeks after implantation, suggesting that the implanted MSCs had spontaneously differentiated into chondrocytes or osteocytes in accordance with their implanted positions (Figure 5).

### Histological examination at 24 and 52 weeks

**Figure 6:**
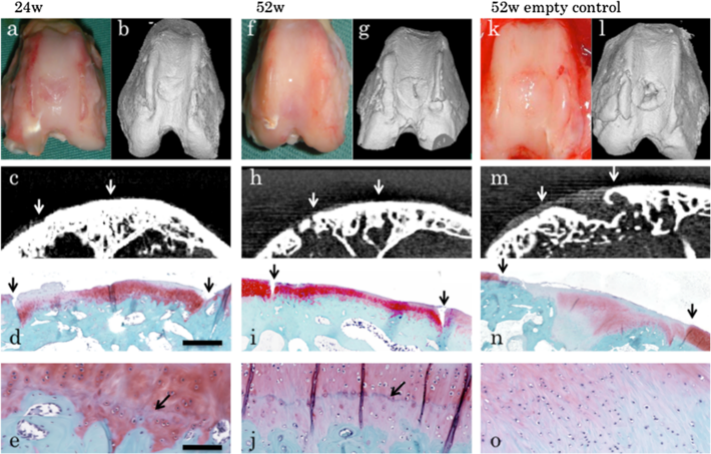
**Morphological and histological examination at 24 and 52 weeks.** Gross appearance, micro-CT scanning, and safranin O staining at **(a–e)** 24 weeks and **(f–j)** 52 weeks. Micro-CT scans revealed complete regeneration of the subchondral bone. Histological examination revealed that the regenerated cartilage was maintained with a consistent thickness identical to that of the surrounding original cartilage at both **(d)** 24 weeks and **(i)** 52 weeks. Scale bar = 1 mm. **(e, j)** Tidemarks, which separate hyaline cartilage from the calcified cartilage, were seen (arrows). Scale bar = 100 μm. **(k–o)** A control rabbit without any implantation at 52 weeks. The defect was mainly filled with fibrous tissue that did not exhibit formation of subchondral bone **(m)** or sufficient staining with safranin O **(n)**. Although some area was stained with safranin O, tidemark was not formed **(o)**. Downwards arrows indicate the borders of the defect. Representative subjects of three experiments for each time course are shown.

**Figure 7:**
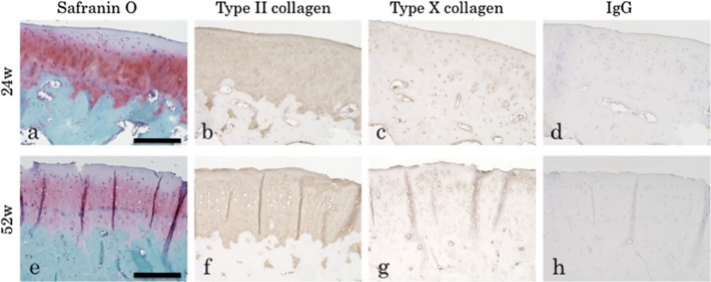
**Immunohistochemistry at 24 and 52 weeks.** Safranin O staining **(a, e)**, immunostaining for type II collagens **(b, f)**, immunostaining for type X collagens **(c, g)**, and IgG staining as controls **(d, h)** at 24 weeks **(a–d)** and 52 weeks **(e–h)**. No positive Immunostaining for type X collagens was observed in the implanted area during the regeneration period. Scale bar =200 μm. Representative subjects of three experiments for each time course are shown.

After obtaining the preliminary results regarding the early regeneration process, we focused on morphological and histological evaluation at 24 and 52 weeks (*n* = 3 each) (Figures 6a–j and 7a–h). CT scans revealed complete regeneration of the subchondral bone. Histological examination revealed that hyaline cartilage, which was stained for safranin O and immunostained for type II collagen, was maintained at a consistent thickness identical to that of the surrounding original cartilage at both 24 and 52 weeks. Immunostaining for type X collagens did not show positive staining in the implanted area during the regeneration period. Tidemark, which separates hyaline cartilage from the calcified cartilage layer, was seen in these two groups, suggesting the integrality of the regenerated articular structures. By contrast, a control rabbit without any implantation showed that the defect was mainly filled with fibrous tissue that did not exhibit the consistent staining for safranin O or formation of the subchondral bone. Although some area was stained with safranin O, tidemark was not formed (Figure 6k–o).

The mean histological score was 10 ± 1.7 (standard error (S.E.)) in the 24-week group and 9.7 ± 0.6 (S.E.) in the 52-week group. There was no significant difference between the 24- and 52-week groups in either parameter of the score, indicating that no deterioration of the repaired tissue took place during this period (Table 1).

**Table 1:** **Histological scores at 24 and 52 weeks after implantation**

	**24 weeks (** ***n*** **=3)**	**52 weeks (** ***n*** **=3)**	***p*** **value**
Cell morphology	2.7 (2–3)	2.7 (2–3)	N.S.
Matrix staining (metachromasia)	1.7 (1–2)	1.7 (1–2)	N.S.
Surface regularity	3	3	N.S.
Thickness of cartilage	1.3 (1–2)	1.7 (1–2)	N.S.
Integration of donor to adjacent host cartilage	1.3 (1–2)	0.7 (0–1)	N.S.
Total score (score range 0–14)	10 (8–11)	9.7 (9–10)	N.S.

*N.S.* not significant.

## Discussion

In this study, we demonstrated successful regeneration of articular cartilage and subchondral bone in an osteochondral defect of rabbit knee joints using three-dimensional HDMACs. The HDMACs were made without using any differentiation agents, such as growth factors, and were implanted to the osteochondral defect while the cells in the construct were undifferentiated. The implanted MSCs spontaneously differentiated into bone or cartilage, depending on the environment in which they were implanted, and maintained the original border between bone and cartilage. Eventually, by the 52-week follow-up, the simultaneous regeneration of bone and cartilage had been completed. The unique features of our method are as follows: 1) A three-dimensional MSC construct with sufficient thickness for bone and cartilage repair can be made without a scaffold. 2) The construct is implanted without induction of differentiation. 3) Simultaneous regeneration of bone and cartilage occurs spontaneously by reproducing the differentiation process involved in bone and cartilage formation. A major strength of our study is that we could confirm that the regenerated bone and cartilage were maintained for up to 52 weeks.

However, one limitation of our study is that we did not make an effort to increase the sample sizes for the short-term results (e.g., within 12 weeks). After confirming in a few samples that MSCs spontaneously differentiate into bone or cartilage *in vivo*, as we originally hypothesized, we placed greater emphasis on investigating the long-term results. We chose this course because a large number of previous studies using other methods had succeeded over the short term, but it remained that deterioration of the repaired cartilage would occur over long-term observation. Some previous studies using a scaffold reported frequent invasion of a bony structure into the repaired cartilage, causing the cartilage to become thinner than the surrounding normal articular cartilage [9,17]. Furthermore, a scaffold has the potential to influence surrounding microenvironments, which can affect the regional specification of implanted MSCs [10]. The presence of nonphysiological scaffolds could interfere with the physiological distribution of signal molecules, such as growth factors, that influence the differentiation of cells toward bone and cartilage.

Following a report of the efficacy of ACI by Brittberg et al. [4], chondrocytes have been widely used in the field of articular cartilage regeneration. The form of the grafts used for implantation varies from monolayer culture [4] to tissue-engineered three-dimensional cultures in agarose [18] or atelocollagen gel [19]. A number of clinical trials have been conducted, and some of them reported successful outcomes in regard to patients’ symptoms or imaging endpoints [19,20]. However, it should be noted that ACI was more effective in patients younger than 45 years than in older patients [21]. In fact, the mean age of clinical trials with satisfactory outcomes for relatively long follow-up periods ranges from 26.4 to 33.1 years old [19,22]. This result may be related to the shortening of telomeres in many adult cells, including chondrocytes [23]. Therefore, it may be necessary to use alternative methods in middle-aged and elderly patients.

Bone and cartilage have the same origin, i.e., MSCs. In a previous work, patient age made no difference in outcomes of methods using MSCs [21]. Therefore, MSCs represent an attractive alternative to adult chondrocytes as a cell source for bone and cartilage regeneration. However, it is challenging to artificially control differentiation into the osteocyte or chondrocyte lineage after implantation. The underlying molecular mechanism of our method, in which a bone and articular cartilage defect was repaired while maintaining the border between cartilage and subchondral bone, is currently unclear. However, cell tracking analysis revealed that both cell types in the repaired tissues were derived dominantly from the implanted MSCs within the construct rather than the migrated cells from the surrounding tissues (Figure 5). Our HDMACs may mimic the cellular integration of embryonic osteogenesis and chondrogenesis that originates from mesenchymal condensation. The HDMACs were implanted without pretreatment to induce differentiation. The micrographs by SEM revealed that the HDMACs contained flattened cells, not ovary-shaped chondrocyte-like cells (Figure 2). Combined with the fact that the HDMACs were not stained with safranin O (Figure 1d), these cells are suggested to be undifferentiated. On the other hand, we also confirmed that the constructs could undergo differentiation into chondrocytes *in vitro* when cultured in chondrocyte induction medium (data not shown). *In vivo*, the implanted MSCs spontaneously differentiate into articular cartilage and subchondral bone; eventually, the normal structures of bone and cartilage were completely formed (Figures 6 and 7). In addition, type X collagen was not expressed in the implanted area. This suggests that hypertrophy of articular cartilage, one of the major problems using MSC-based constructs, did not occur during the regeneration period.

Our study is distinguished not only by the use of undifferentiated MSCs but also by the use of a construct generated by a scaffold-free method. Our HDMACs have enough volume to fill a full-thickness cartilage defect. Cell nutrition particularly in the middle of a big construct can be a concern [24]. However, the nucleus was stained with hematoxylin in the middle of the HDMACs and there was no fragmentation or chromatin condensation (Figure 1d). This suggests that the cells in the construct were viable. Previous studies developed various scaffold-free methods using chondrocytes or MSCs; the simplest reported method is injection of synovial MSCs into a cartilage defect *in vivo* [16]. In that study, however, although the histological score was significantly greater than the control, the depth of the defect was restricted to 2 mm and the surface was concave. Another method using a scaffold-free construct derived from synovial MSCs resulted in successful repair of a cartilage defect [14], but again, the depth was limited to 2 mm. In yet another study, scaffold-free chondrocyte sheets were used successfully to repair of a partial-thickness cartilage defect *in vivo* [13]. However, this method was insufficient for subchondral bone repair and tissue integration in the context of a full-thickness cartilage defect [25]. In efforts to obtain enough cell mass to fill a full-thickness cartilage defect, pellet cultures of chondrocytes or MSCs have been investigated. In one animal study, chondrocyte pellet implantation into a 3-mm osteochondral defect was attempted [15]. Although the thickness of the repaired cartilage remained relatively constant, overgrowth of the surface was observed, and subchondral bone regeneration was extremely limited.

## Conclusions

Our novel technique, using a three-dimensional scaffold-free autologous construct derived from BM-MSCs, successfully achieved the simultaneous regeneration of bone and cartilage for up to 1 year *in vivo*. This method has potential for clinical use as a safe and effective method for repairing bone and cartilage defects.

## References

[1] Buckwalter JA, Mankin HJ (1998). Articular cartilage repair and transplantation. Arthritis Rheum.

[2] Matsusue Y, Yamamuro T, Hama H (1993). Arthroscopic multiple osteochondral transplantation to the chondral defect in the knee associated with anterior cruciate ligament disruption. Arthroscopy.

[3] Steadman JR, Briggs KK, Rodrigo JJ (2003). Outcomes of microfracture for traumatic chondral defects of the knee: average 11-year follow-up. Arthroscopy.

[4] Brittberg M, Lindahl A, Nilsson A (1994). Treatment of deep cartilage defects in the knee with autologous chondrocyte transplantation. N Engl J Med.

[5] Kreuz P, Steinwachs M, Erggelet C, Krause S, Konrad G, Uhl M, Sudkamp N (2006). Results after microfracture of full-thickness chondral defects in different compartments in the knee. Osteoarthritis Cartilage.

[6] Bentley G, Biant LC, Carrington RWJ, Akmal M, Goldberg A, Williams AM, Skinner JA, Pringle J (2003). A prospective, randomised comparison of autologous chondrocyte implantation versus mosaicplasty for osteochondral defects in the knee. J Bone Joint Surg (Br).

[7] Knutsen G, Engebretsen L, Ludvigsen TC, Drogset JO, Grøntvedt T, Solheim E, Strand T, Roberts S, Isaksen V, Johansen O (2004). Autologous chondrocyte implantation compared with microfracture in the knee. A randomized trial. J Bone Joint Surg Am.

[8] Wakitani S, Kimura T, Hirooka A, Ochi T, Yoneda M, Yasui N, Owaki H, Ono K (1989). Repair of rabbit articular surfaces with allograft chondrocytes embedded in collagen gel. J Bone Joint Surg (Br).

[9] Wakitani S, Goto T, Pineda SJ, Young RG, Mansour JM, Caplan AI, Goldberg VM (1994). Mesenchymal cell-based repair of large, full-thickness defects of articular cartilage. J Bone Joint Surg Am.

[10] Koga H, Muneta T, Ju YJ, Nagase T, Nimura A, Mochizuki T, Ichinose S, Mark von der K, Sekiya I (2007). Synovial stem cells are regionally specified according to local microenvironments after implantation for cartilage regeneration. Stem Cells.

[11] Anderson JM, Rodriguez A, Chang DT (2008). Foreign body reaction to biomaterials. Semin Immunol.

[12] Badylak SF, Gilbert TW (2008). Immune response to biologic scaffold materials. Semin Immunol.

[13] Kaneshiro N, Sato M, Ishihara M, Mitani G, Sakai H, Mochida J (2006). Bioengineered chondrocyte sheets may be potentially useful for the treatment of partial thickness defects of articular cartilage. Biochem Biophys Res Commun.

[14] Ando W, Tateishi K, Hart DA, Katakai D, Tanaka Y, Nakata K, Hashimoto J, Fujie H, Shino K, Yoshikawa H, Nakamura N (2007). Cartilage repair using an in vitro generated scaffold-free tissue-engineered construct derived from porcine synovial mesenchymal stem cells. Biomaterials.

[15] Cheuk Y-C, Wong MW-N, Lee K-M, Fu S-C (2011). Use of allogeneic scaffold-free chondrocyte pellet in repair of osteochondral defect in a rabbit model. J Orthop Res.

[16] Nakamura T, Sekiya I, Muneta T, Hatsushika D, Horie M, Tsuji K, Kawarasaki T, Watanabe A, Hishikawa S, Fujimoto Y, Tanaka H, Kobayashi E (2012). Arthroscopic, histological and MRI analyses of cartilage repair after a minimally invasive method of transplantation of allogeneic synovial mesenchymal stromal cells into cartilage defects in pigs. Cytotherapy.

[17] Adachi N, Sato K, Usas A, Fu FH, Ochi M, Han CW, Niyibizi C, Huard J (2002). Muscle derived, cell based ex vivo gene therapy for treatment of full thickness articular cartilage defects. J Rheumatol.

[18] Benya P (1982). Dedifferentiated chondrocytes reexpress the differentiated collagen phenotype when cultured in agarose gels. Cell.

[19] Takazawa K, Adachi N, Deie M, Kamei G, Uchio Y, Iwasa J, Kumahashi N, Tadenuma T, Kuwata S, Yasuda K, Tohyama H, Minami A, Muneta T, Takahashi S, Ochi M (2012). Evaluation of magnetic resonance imaging and clinical outcome after tissue-engineered cartilage implantation: prospective 6-year follow-up study. J Orthop Sci.

[20] Adachi N, Ochi M, Deie M, Nakamae A, Kamei G, Uchio Y, Iwasa J (2014). Implantation of tissue-engineered cartilage-like tissue for the treatment for full-thickness cartilage defects of the knee. Knee Surg Sports Traumatol Arthrosc.

[21] Nejadnik H, Hui JH, Feng Choong EP, Tai B-C, Lee EH (2010). Autologous bone marrow-derived mesenchymal stem cells versus autologous chondrocyte implantation: an observational cohort study. Am J Sports Med.

[22] Peterson L, Minas T, Brittberg M, Lindahl A (2003). Treatment of osteochondritis dissecans of the knee with autologous chondrocyte transplantation: results at two to ten years. J Bone Joint Surg Am.

[23] Martin JA, Klingelhutz AJ, Moussavi-Harami F, Buckwalter JA (2004). Effects of oxidative damage and telomerase activity on human articular cartilage chondrocyte senescence. J Gerontol A Biol Sci Med Sci.

[24] Rouwkema J, Koopman B, Blitterswijk C, Dhert W, Malda J (2010). Supply of nutrients to cells in engineered tissues. Biotechnol Genet Eng Rev.

[25] Vasara AI, Hyttinen MM, Lammi MJ, Lammi PE, Långsjö TK, Lindahl A, Peterson L, Kellomäki M, Konttinen YT, Helminen HJ, Kiviranta I (2004). Subchondral bone reaction associated with chondral defect and attempted cartilage repair in goats. Calcif Tissue Int.

